# Supplementary material for: Indigenous Australian household structure: a simple data collection tool and implications for close contact transmission of communicable diseases
Source: PeerJ. 2017 Oct 26;5:e3958. doi: 10.7717/peerj.3958 (PMC5660877; doi:10.7717/peerj.3958)
Supplement: Supplemental Information 2 [file peerj-05-3958-s002.pdf]

Indigenous Australian household structure: a simple data collection tool and implications for close contact transmission of communicable diseases

Thiripura Vino[1], Gurmeet Singh[2,3], Belinda Davison[2], Patricia Therese Campbell[5,8], Michael John Lydeamore[1,5], Andrew Robinson[1,6,7], Jodie McVernon[8], Steven Y. C. Tong[2,9], and Nicholas Geard[4,10]

[1]School of Mathematics and Statistics, University of Melbourne, Victoria, Australia
[2]Menzies School of Health Research, Charles Darwin University, Darwin, Northern Territory, Australia
[3]NT medical Program of Flinders and James Cook Universities
[4]Melbourne School of Population and Global Health, University of Melbourne, Victoria, Australia
[5]Murdoch Childrens Research Institute, The Royal Children's Hospital, Melbourne, Victoria, Australia
[6]School of BioSciences, University of Melbourne, Victoria, Australia
[7]Centre of Excellence for Biosecurity Risk Analysis, Victoria, Australia
[8]Victorian Infectious Diseases Reference Laboratory, The Royal Melbourne Hospital and The University of Melbourne, at the Peter Doherty Institute for Infection and Immunity, Victoria, 3000, Australia
[9]Victorian Infectious Disease Service, The Royal Melbourne Hospital, and The University of Melbourne, at the Peter Doherty Institute for Infection and Immunity, Victoria, Australia
[10]School of Computing and Information Systems, University of Melbourne, Victoria, Australia

Corresponding author:
Steven Y. C. Tong

Email address: Steven.Tong@mh.org.au

ABSTRACT

Households are an important location for the transmission of communicable diseases. Social contact between household members is typically more frequent, of greater intensity, and is more likely to involve people of different age groups than contact occurring in the general community. Understanding household structure in different populations is therefore fundamental to explaining patterns of disease transmission in these populations. Indigenous populations in Australia tend to live in larger households than non-Indigenous populations, but limited data is available on the structure of these households, and how they differ between remote and urban communities. We have developed a novel approach to the collection of household structure data, suitable for use in a variety of contexts, which provides a detailed view of age, gender, and room occupancy patterns in remote and urban Australian Indigenous households. Here we report analysis of data collected using this tool, which quantifies the extent of crowding in Indigenous households, particularly in remote areas. We use this data to generate matrices of age-specific contact rates, as used by mathematical models of infectious disease transmission. To demonstrate the impact of household structure, we use a mathematical model to simulate an influenza-like illness in different populations. Our simulations suggest that outbreaks in remote populations are likely to spread more rapidly and to a greater extent than outbreaks in non-Indigenous populations.

1. Household Size distribution for Australian Bureau of Statistics Census data (2011) for the towns selected in ABC study

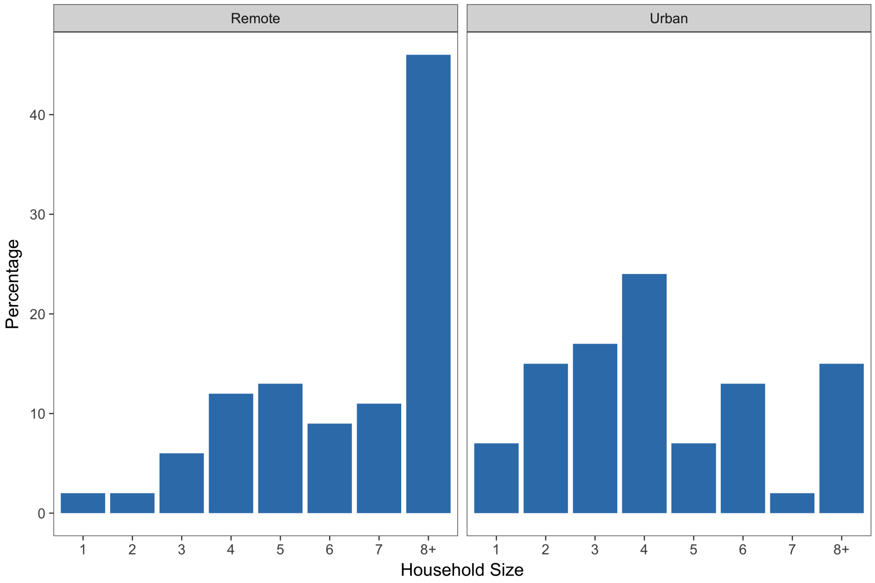

Figure 1. **Household Size distribution for Census data**

2. Contact matrices for each shire

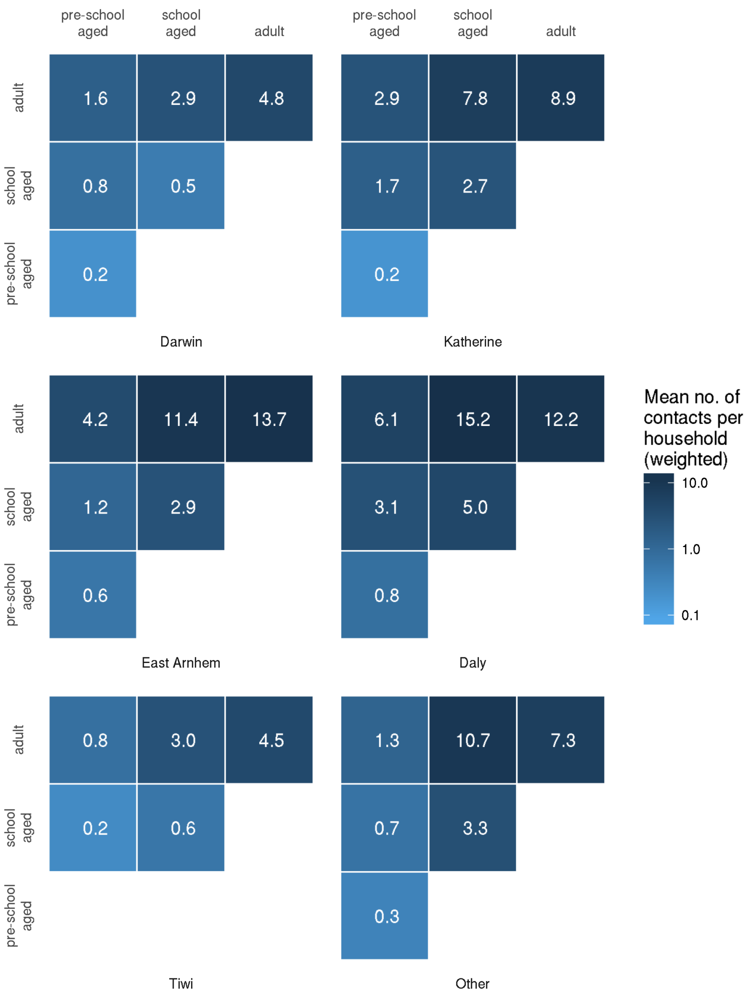

Figure 2. **Household Contact matrices for shires**

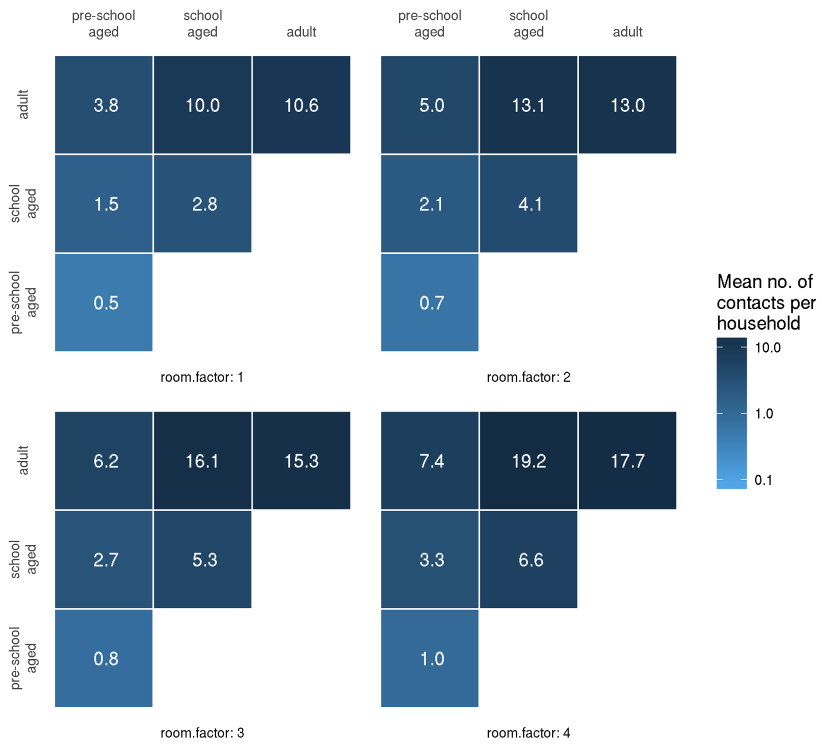

Figure 3. **Effect of weighting by rooms on contact matrices**
