# Supplementary material for: Indigenous Australian household structure: a simple data collection tool and implications for close contact transmission of communicable diseases
Source: PeerJ. 2017 Oct 26;5:e3958. doi: 10.7717/peerj.3958 (PMC5660877; doi:10.7717/peerj.3958)
Supplement: Supplemental Information 4 — Information brochure for participants in the Aboriginal Birth Cohort Study [file peerj-05-3958-s004.pdf]

Frequently asked questions

What happens to the information?

The information you give us is protected and kept in locked files at Menzies School of Health Research and only available to researchers on this project. The results will be fed back to communities, health professionals and other researchers.

Is my information confidential?

All of the information you give us will be kept private and you will not be identified.

How long does it take?

Completing all of the procedures will take about 1½ to 2 hours.

What do I need to know?

We will explain each test in detail when we meet and then ask you to sign a consent form if you are happy to take part in the study. We will also explain each procedure while you are doing it. We are happy to answer any questions at the clinic or afterwards.

Do I have to answer all of the questions?

No. You can choose to answer which questions you feel comfortable with.

What happens with my blood and urine samples?

The blood and urine tests are done in laboratories interstate e.g. Adelaide and Brisbane. This means that, with your consent, we will store your blood and urine in freezers at Menzies until sent to the laboratories. It also will mean that there is likely to be a delay before you get all of the results.

You can talk more about this with

Belinda Davison
ABC Project manager
Menzies School of Health Research

Phone: (08) 8922 8701 or 0458 543 765
Email: belinda.davison@menzies.edu.au

If you have any concerns or complaints regarding the ethical conduct of the study, you are invited to contact Ethics Administration, Human Research Ethics Committee of the Northern Territory Department of Health and Menzies School of Health Research on

(08) 8922 7922 or (08) 8922 8705 or email ethics@menzies.edu.au

menzies
school of health research
discovery for a healthy tomorrow

PO Box 41096 Casuarina NT 0811 Australia
Phone: (08) 8922 8196
Fax: (08) 8927 5187
Email: info@menzies.edu.au
Website: menzies.edu.au
Menzies School of Health Research (HQ)
John Matthews Building (JMB)
Building 58, Royal Darwin Hospital Campus
Northern Territory, Australia 0810

menzies
school of health research

ABC STUDY

Aboriginal Birth Cohort Study

Across the life course 1987 to 2013 and beyond

What is the ABC Study?

When you were born in 1987-1990 at Royal Darwin Hospital, your mum gave permission for you to be part of a life course study, the Aboriginal Birth Cohort (ABC) study.

You, along with over 680 other children born at the same time are now part of the longest and largest prospective cohort study of Aboriginal people in Australia.

Since your birth we have seen you, in your community, at age 11 and 18 years and assessed your health.

Now that you are young men and women in your mid 20s the ABC research team is looking forward to meeting you and all the other participants in 2013 or 2014.

The aim of this study is to relate life events, beginning in the womb, to later physical and mental health.

This will help identify early those most at risk of: developing chronic diseases such as

- **diabetes**
- **cardiovascular (heart)**
- **renal (kidney)**
- **and mental health**

It will also help develop strategies to contribute to closing the current gap in life expectancy between Aboriginal and non-Aboriginal Australians.

Why do we want to do this?

We want to find out if babies born small or too early grow up differently to babies who are born at term and with normal birth weights. Some studies in other countries have shown that being born early or small can affect your adult health.

We know that too many Aboriginal people die before they are old from problems with heart disease, kidney disease, lung problems and diabetes.

Poor adult health may also be affected by living conditions, emotional well-being and physical health of children as they grow up.

We want to look at how these things affect your health as you grow up and get older.

Answers to these research questions will help us plan better ways to stop these chronic diseases from occurring and reduce the number of people dying early.

What's involved?

If you agree to continue to be part of this important study we would like to see you at one of our clinics.

At the clinic we will provide you with more information on all items that make up this health check.

Some of the things we offer are

- body measurements
- blood pressure
- ultrasounds of kidneys, thyroid and carotid (artery in your neck)
- blood and urine testing
- emotional well-being and thinking ability

What will you get out of this?

We expect you to be healthy and so your health check may be of no immediate use to you personally.

We will be testing your health and checking for early signs of heart disease, kidney disease and diabetes.

We are also looking to see if the tests suggest that you are at risk for specific health issues later in life.

With your consent, any abnormalities or problems in the areas examined will be sent to your health clinic or GP.

You will be helping researchers gain knowledge that will be of great importance to the future health of the community and you will learn more about your own health.

What are your options?

You can agree to do all of the study and continue to be part of a special group who are helping researchers understand how to prevent disease.

You will then be asked to attend a health check in your community. At the health check you can agree to either

- Do all of the procedures offered
- Choose which procedures you participate in.

You can refuse to be part of this study or withdraw at anytime. This will not affect medical advice in the management of your health, now or in the future.
