# Supplementary material for: Indigenous Australian household structure: a simple data collection tool and implications for close contact transmission of communicable diseases
Source: PeerJ. 2017 Oct 26;5:e3958. doi: 10.7717/peerj.3958 (PMC5660877; doi:10.7717/peerj.3958)
Supplement: Supplemental Information 5 — Information sheet to accompany informed consent forms for the Aboriginal Birth Cohort Study [file peerj-05-3958-s005.doc]

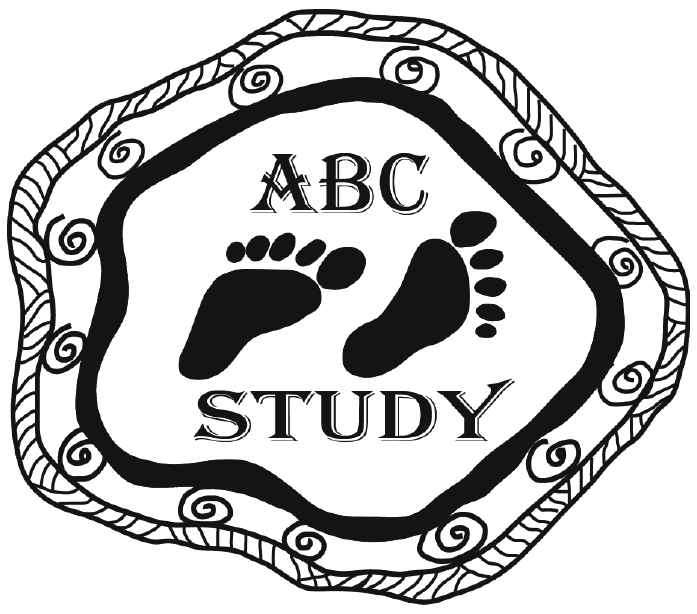


**This is for you to keep**

**INFORMATION SHEET**

Aboriginal Birth Cohort Study: Wave-4

**Background**

You are part of special study called The **Aboriginal Birth Cohort Study**. In 1987-1990 your mother agreed for you to be part of this exciting study examining the health and wellbeing of Aboriginal babies born in the Top End. We have already assessed you when you were aged 11 years and 18 years. We would like to again thank you for being part of this study and invite you to participate in this wave of the study.

The aim of this study is to monitor your health over your life span. Obesity has reached epidemic proportions in Australia and Type 2 diabetes rates are increasing with earlier age of onset. These and other chronic conditions including heart disease and kidney disease can increase the risk of dying at an earlier age than expected.

Information we gain from this study and others like it allows researchers to detect risk factors for adult disease which if treated early can improve life expectancy.

When you were last seen by the research team we asked your permission to contact you again in 4-5 years. We are now asking your permission to continue to participate in the study and to again attend a health check clinic.

**What’s involved?**

If you consent to continuing to be part of this important study we would like to see you at one of our clinics. These clinics will be almost the same as the last clinic you attended and will be held in your community.

At the clinic we will provide you with further information on all items of the health check. Although we would like you to participate in all activities, **remember it is your choice**. You can choose to either participate in all of them or choose which you would like to participate in.

As in the last clinic we would like to do body measurements, including your height and weight, head, arm, waist and hip measurements.


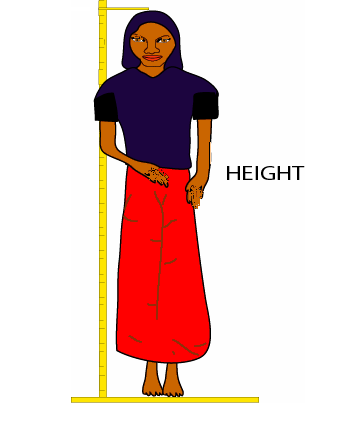

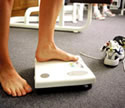

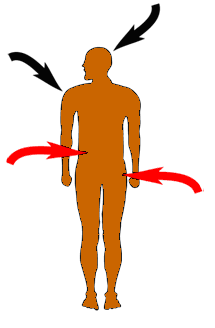


We would also like to take your blood pressure and heart rate.


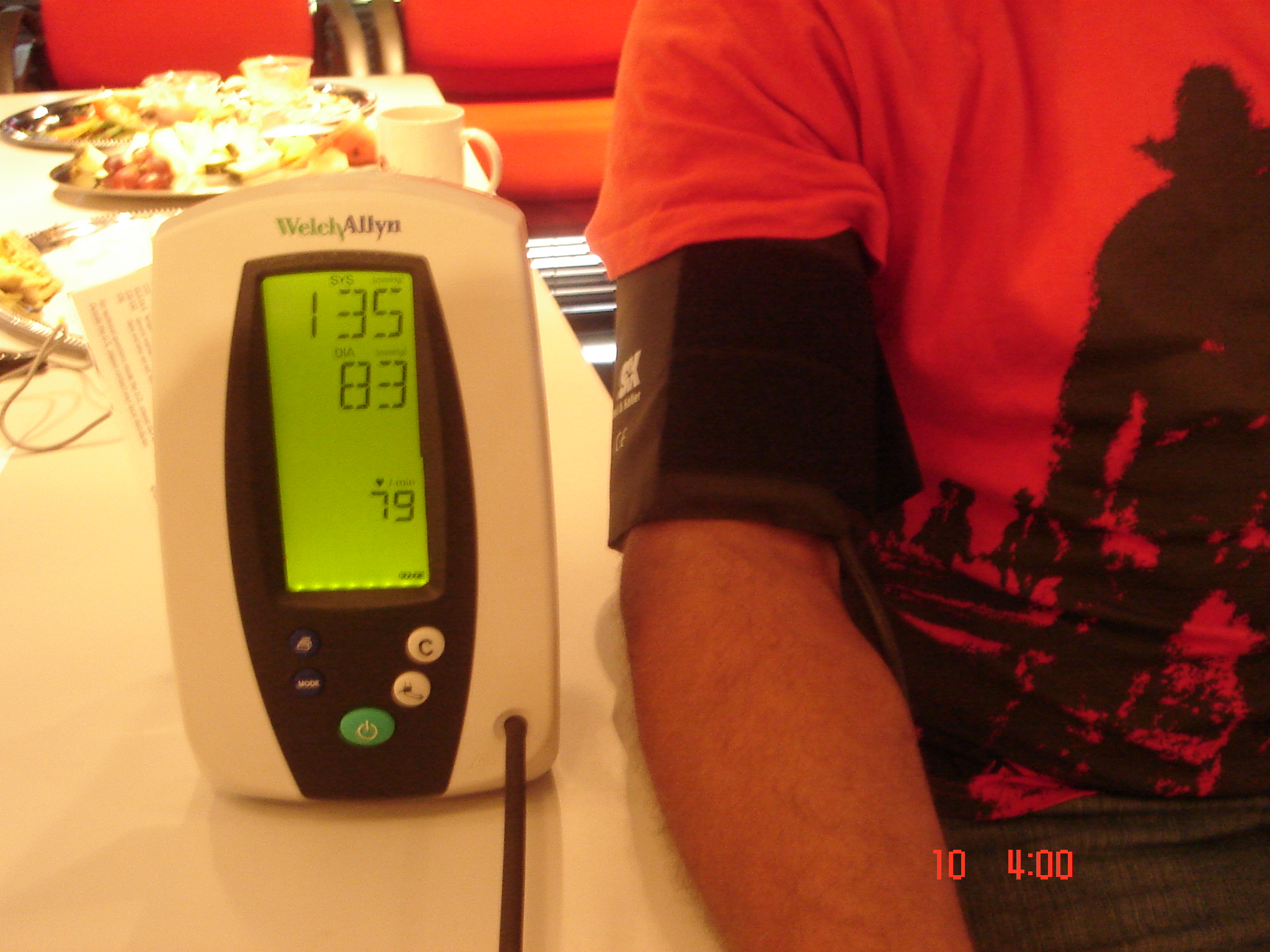

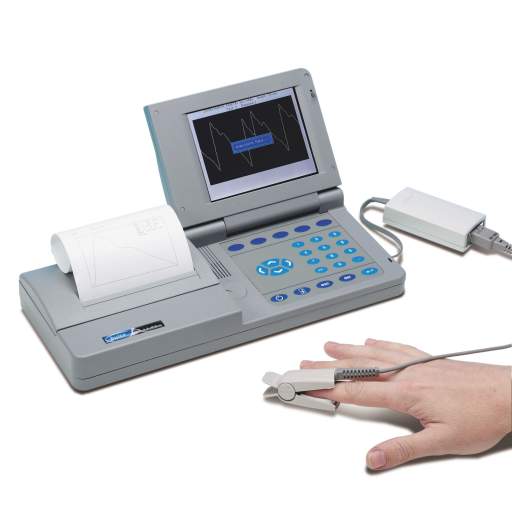


Also Dr Gurmeet Singh will again do ultrasounds of your kidney, thyroid and carotid.


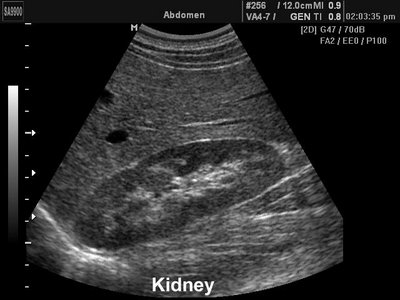


We would also like to collect and examine a small amount of urine and blood. This means you should not have anything to eat or drink on the morning of the clinic. We will provide food and fruit juice after the blood is taken. The blood will be taken in sterile conditions and care will be taken not to hurt you. What we do with your blood and urine is explained below.

**We can put on cream that numbs the skin.**

We would also ask that you complete a checklist about your emotional wellbeing and do a computer game to test your memory. You can also see if your grip strength has improved (or not).


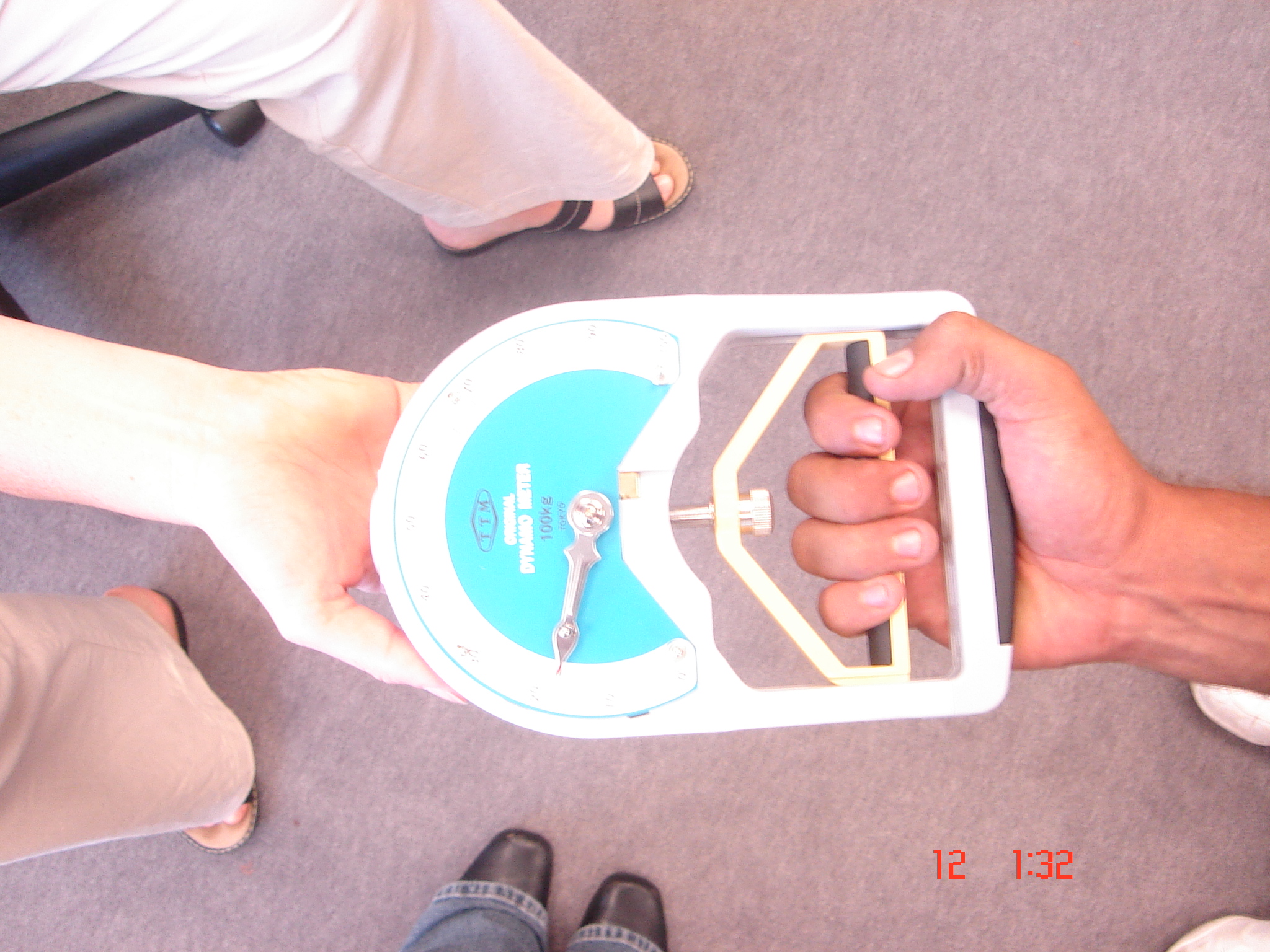


This time, as we did when you were 11, we would also like to check your lung function


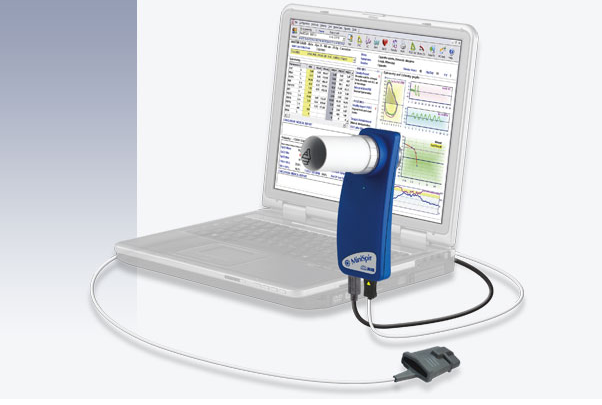


The complete check-up takes about 1.5 to 2 hours.

With your permission we would like to take a digital picture of you that may later be used on posters, on our website and\or in our photo albums.

**Why do we want to do this?**

We want to try and find out if babies born too small or babies born too early grow up differently to babies who are born at the right time and the right size. We know that too many Aboriginal people die before they are old from heart problems, kidney disease, lung problems and diabetes. Some studies in other countries show adult health may be directly related to the size of the baby when it is born. Other people believe adult health may be due to living conditions, emotional well-being and physical health of the children as they grow up. That is why we recruited you at birth so we could look at your birth records to see how big you were as a baby.

If we can get the answers to these research questions we can plan ways to prevent these chronic diseases and reduce the number of people dying early.

**How will we look after the information and the samples**?

The data collected is recorded on sheets kept in locked files at the Menzies School of Health Research and entered into computer programs. Information is only available to the research team and is password protected. When analysing the study data only study numbers are used. As this is a longitudinal study, information collected will be kept for use in later waves of this study. It will be compared with your last lot of results and any future results.

For most of the blood and urine tests we will have to use laboratories based other than Darwin, e.g. Adelaide and Brisbane. Due to transport costs this means that the tests will be done in batches. What this means is that, with your consent, we will store your blood and urine in freezers at Menzies until sent to the laboratories. It also will mean that there is likely to be a delay before you get all the results.

Publications about this work will be made without any identifying facts relating to individuals or their community. We are developing a website where details of the study and any outcomes will be published. You will be informed on how to access this at your clinic visit.

**What will you get out of this?**

From this health check we will be able to check your growth, nutritional status, blood pressure and general health. We are also assessing if you are at risk for heart disease or diabetes in later life. We will check your emotional well-being and thinking ability. With your consent, any abnormalities or problems in the areas examined will be sent to your nominated health clinic or GP.

You may not get that much out of this study personally but you will be helping researchers gain knowledge that will help the future health of the community and you will learn more about your own health.

**What are your options?**

1. You can agree to do all of the study and continue to be part of a special group who are helping researchers understand how to prevent disease
2. You can agree to be part of the study but chose which parts you participate in and continue to be part of a special group who are helping researchers understand how to prevent disease
3. You can refuse to be part of this study or withdraw at anytime. This will not affect medical advice in the management of your health, now or in the future.

**You can talk more about this with:**

Belinda Davison, project manager

Menzies School of Health Research

Phone 8922 8701 or email [belinda.davison@menzies.edu.au](mailto:belinda.davison@menzies.edu.au)

Or you can visit the website at [www.menzies.edu.au/topendcohort](http://www.menzies.edu.au/topendcohort)

If you have any concerns or complaints regarding the ethical conduct of the study, you are invited to contact the Ethics Administration, Human Research Ethics Committee of the Northern Territory Department of Health and Menzies School of Health Research on

(08) 89227922 or (08) 89228705 or email [**ethics@menzies.edu.au**](mailto:ethics@menzies.edu.au)

**This information sheet is for you to keep**
